# Laboratory Study on Injection Force Measurement on Syringe and Needle Combinations

**DOI:** 10.21315/mjms2019.26.2.8

**Published:** 2019-04-30

**Authors:** Theddeus Octavianus Hari Prasetyono, Prasasta Adhistana

**Affiliations:** 1 Division of Plastic Surgery, Department of Surgery, Cipto Mangunkusumo Hospital/Faculty of Medicine Universitas Indonesia, Jakarta, Indonesia; 2 ICTEC (Indonesian Clinical Training & Education Centre), Cipto Mangunkusumo Hospital/Faculty of Medicine Universitas Indonesia, Jakarta, Indonesia; 3 Central Pertamina Hospital, Jakarta, Indonesia

**Keywords:** local anesthesia, injections, plastic surgery, tumescent

## Abstract

**Background:**

This study aimed to measure the least initial and maintenance forces of syringe and needle combinations to provide a reference for local anesthetic injection.

**Methods:**

An experimental study was conducted in our Physics Laboratory during September 2015. A series of syringes sized 1 mL, 3 mL, 5 mL, 10 mL and 20 mL were paired with the original needles, 27G, 27G spinal and 30G. Each combination was tested three times using a compression testing Instron 5940 Series to measure initial and maintenance forces. Statistical analysis was performed using One-way ANOVA.

**Results:**

The lowest initial force was shown by the combination of 1 mL syringe and 27G spinal needle. However, the 1 mL syringe showed no significant difference across the needles [*F*(3, 8) = 3.545; *P* < 0.068]. The original and 27G needle showed mean difference 0.28 (95%CI: −0.19, 0.75; *P* = 0.420). The lowest maintenance force was measured in the combination of 1 mL syringe and its original 26G needle. On the contrary, both the highest initial and maintenance forces were shown by the combination of 10 mL syringe and 30G needle.

**Conclusion:**

The 1 mL syringe with original 26G needle shows the best combination.

## Introduction

Injection into the volar side of the hand and digit may be considered as one of the most painful local injection; thus, it could be a good model to discuss the idea of providing the least pain possible while local anesthesia injection. The tumescent solution, which contains local anesthetic epinephrine and lidocaine, has been practiced by surgeons to facilitate FAHS (full awake hand surgery) (1). FAHS needs not to apply a pneumatic tourniquet, which obviously needs general anesthesia. Local infiltrative, as well as tumescent anesthesia injection, are also practiced widely for many surgical procedures, including in finger (2).

Besides creating a clear operative field, FAHS is expected to be pain-free throughout the surgery (3). Technically, pain sensation starts when the first needle punctures the skin. It may also be elicited while injecting the amount of anesthesia solution under tumescent concept; especially if it is injected too fast. One common method used to minimise the pain caused by needle insertion is to use a small-sized needle. Several syringes can be used to deliver the injection; ranging from 1 mL to 20 mL syringe (4, 5). Utilisation of 20 mL syringe and 27G needle with “hole-in-one” principle is said to minimise the pain (6). However, this technique may potentially create painful sensation because the amount of solution delivered cannot be meticulously controlled due to the large force needed to pass the solution through a small needle calibre. The strong force effect along with the steadiness of the personnel injecting the solution would physically complicate the pain stimulation. Many doctors use a 1 mL syringe and 27G needle to lower the resistance created when the amount of solution has to pass through a small needle. Hence, the power or force needed to expel the solution is much lower (7).

While the needle geometry and the surface smoothness of the syringe affect injectability (7), it is hypothetically the matched-pair of the syringe and needle calibre that matters. Injection of solution requires two types of force as the parameters of injectability, i.e. (i) the initial force when the piston of the syringe is pushed; known as plunger-stopper break loose force (PBF) and (ii) the maintenance force required to keep pushing the piston in a sustained way; known as dynamic gliding force (DGF) (8, 9). Both forces are affected by the diameter of the needle and syringe, as well as the viscosity of the solution. However, the best combination of syringe and needle required to establish the least force needed to inject and the least pain for patients is still unknown. Therefore, we aimed to study the physical and mechanical aspect regarding the power and force in the combination of needle and syringe used for local anesthesia injection before studying it directly in human subjects.

## Materials and Methods

**Figure 1:**
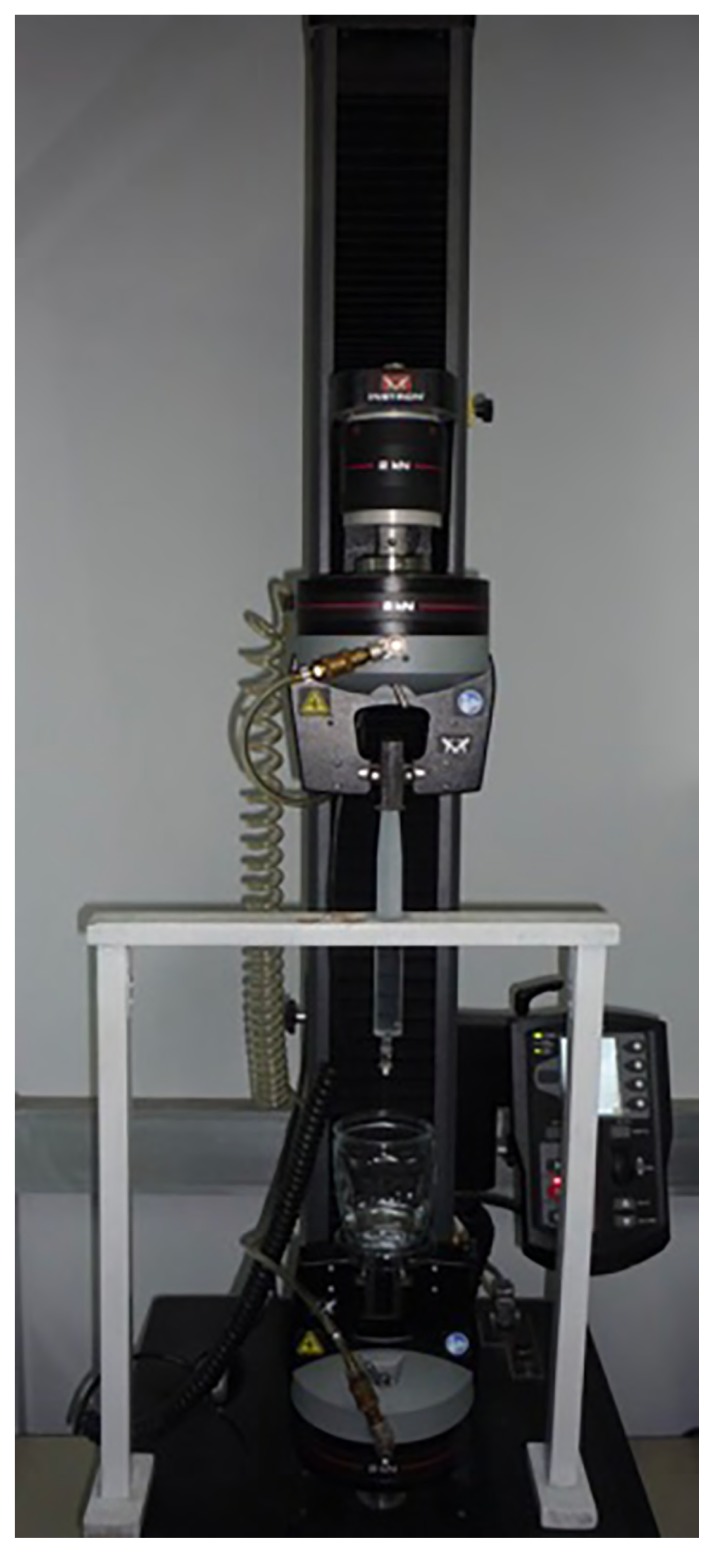
The Instron 5940 device is a single column tabletop model testing system to measure the initial and the maintenance force of the syringe and needle pairs

An experimental study was conducted in Physics Laboratory of Faculty of Medicine, Universitas Indonesia using Instron 5940 series (Instron, Norwood, USA) to get data on force value of the injection kit model. Instron 5940 series (Figure 1) is a single column tabletop testing system to measure the initial force known as PBF and the maintenance force known as DGF as the parameters of injectability. It can be used over a range of force applications up to 2 kN (450 lb or 200 kg).

Twenty combinations of syringe and needle, (Table 1 Suppl), were tested to study the physical and mechanical aspect regarding power and force. Each combination was tested using Instron 5940 series testing systems in 100 mm/min velocity. The syringes used were 1 mL, 3 mL, 5 mL, and 10 mL with the original needles from the packaging (Terumo Corporation, Tokyo, Japan), respectively. The 1 mL syringe originally pairs with a 26G needle; the 3 mL with 23G, 5 mL with 22G and the 10 mL with 21G. Only the 20 mL syringe did not come with an original needle pair. All syringes were also combined with a 27G needle, 30G needle (Terumo Corporation, Tokyo, Japan), and 27G spinal needle (B Braun Medical Inc, Melsungen, Germany). Combinations of the syringes and needles were tested three times in the machine.

Parameters of injectability were PBF and DGF for a given needle-syringe combination. Every combination was expressed as the mean (SD) value of triplet measurements. SPSS version 22.0 was used to process the data. The normality was tested using Kolmogorov Smirnov. Finally, the data was analysed using one way ANOVA test.

## Results

The highest initial force (PBF) of the 1 mL syringe was 1.15 (SD 0.22) N by the original combination with the 26G needle (Table 2 Suppl). To a surprise, the lowest PBF of 1 mL syringe was in combination with the longest needle studied, 27G spinal needle [0.75 (0.087) N]. By contrast, the highest maintenance force (DGF) in 1 mL syringe was achieved when combined with a 30G needle [0.71 (0.06) N]; meanwhile, the lowest was with the original 26G needle [0.13 (0.05) N]. As shown in Table 1, there is no significant difference between needles for PBF. However, the DGF showed a significant difference between needles, except the 27G and 27G spinal needles.

For 20 mL syringe, combination with 30G needle failed the trial due to too much pressure passing through the needle. The combination with 27G needle showed the highest PBF [28.33 (1.44) N] as well as the DGF [134.53 (0.61) N], while the lowest PBF was achieved by the combination with 27G spinal needle [25.33 (0.578) N]. Interestingly, the lowest DGF was achieved also by the same combination with a 27G spinal needle [113.27 (1.55) N]. Statistically, the outcome of the tests for the 20 mL syringe was not easily determined, since there was no original needle pair in its packaging to compare. The results of the combination with a 27G needle and 27G spinal needles were significant for both PBF and DGF (Table 1).

The statistical analysis for syringes with respective original needles shows that there was no significant difference in PBF between 3 mL and 5 mL syringes. In terms of the DGF, 5 mL and 10 mL syringes show no significant difference (Table 2).

**Figure 2:**
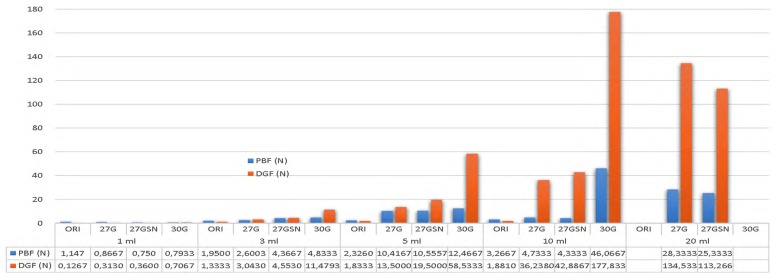
Means of PBF and DGF Syringes include 1 mL, 3 mL, 5 mL, 10 mL, and 20 mL paired with their original needles as well as 27G, 27G spinal, and 30G needles DGF (dynamic glide force), N (Newton), ORI (original needle from packaging of the syringe), PBF (plunger-stopper break-loose force), 27GN (27-Gauge needle), 27GSN (27-Gauge spinal needle), 30GN (30-Gauge needle)

Overall, the lowest PBF value was achieved by the combination of 1 mL syringe and 27G spinal needles; and the highest PBF value was achieved by the combination of a 10 mL syringe and 30G needles. Interestingly, the lowest DGF value was measured in the combination of a 1 mL syringe and its original 26G needle. In accordance with the highest PBF value, a combination of a 10 mL syringe and the 30G needle has the highest DGF value (Figure 2).

## Discussion

The earliest amount of fluid being injected cause pain following the pain caused by the needle puncture during local injection. Therefore, proper attention should be given to the merits of the injection device, which includes the syringe and the needle. This study shows that the overall lowest PBF is achieved by 1 mL syringe combined with a 27G spinal needle. Apparently, this spinal needle has a greater inner diameter (0.28 mm) than the 27G (0.19 mm). In general, length is an important factor that contributes to the energy needed to start flowing fluid into a small pipe (9). However the finding shows that the length of the needle seems not an important factor to cause more restraining force during injection, knowing that the inner diameter of the needle is obviously greater.

Clinically, the moment of pushing the piston sliding inside the syringe takes a crucial role in potential pain created by the flowing solution inside the tissue. The speed of the gliding piston correlates with the flow of the fluid infiltrating the tissue. Greater the speed the more is the stimulation to nerve endings, which means more pain. It is well accepted that slow flowing injection creates less pain than relatively faster injection, especially during the initial millilitres. According to our clinical experiences, the first millilitre is the main volume to be adaptable to the patient’s feeling. Once it settles in 1 min to 5 min to allow the anesthesia to work (10, 11), the second millilitre would be well adapted by most patients. This study interestingly showed that the DGF is best shown by the original 26G needle in its pair with a 1 mL syringe.

Statistically, there was no significant difference in PBF between needles in the study of the 1 mL syringe (Table 1). However, the DGF values were significantly different between the 26G and 30G needles, but not for 27G and 27G spinal needles. As the PBF amongst the needles was not significantly different, it suggests that 1 mL syringe is at best in combination with a 26G needle, which is the original pair. Due to the small capacity of the 1 mL syringe, it is necessary to repeatedly reload the syringe to get the required larger amount of tumescent solution needed in the clinical application. The syringe is usually reloaded without necessarily taking the needle out from and into the tissue repeatedly; or at least with minimum frequency of needle punctures. Nevertheless, the use of the 1 mL syringe is a premium recommendation as it allows us to own better control on the fluid amount and the speed of injection in regard to get a pain free injection.

Besides using small volume syringe to get better volume as well as the speed control to create the least pain in local injections, surgeons also use 20 mL syringe as the 20 mL syringe paired with 27G spinal needle has been reported to work well (10, 12). Surgeons could perform the injection slowly while taking advantage of small needle caliber to minimise pain. Fortunately, this study confirms their practices as both the lowest PBF and DGF in the study of the 20 mL syringe were shown by the 27G spinal needle. The fact was also confirmed by statistical analysis where the differences between 27G and 27G spinal needles were significant, showing 27G spinal needle is a better choice than the 27G (Table 1).

Evidently, in their original pairs, 3 mL syringe and the 23G needle have no difference in PBF with the 5 mL and 22G pair. Likewise, the original pair of 5 mL and 22G performs no differently with the pair of 10 mL and 21G in terms of DGF. This finding informs us that the original pairs of 3 mL, 5 mL and 10 mL syringes are suitable for any situations, regardless of needle piercing related pain. Nonetheless, as it is shown in Figure 2, the 20 mL syringe in either combination with a 27G or 27G spinal needle is not comparable to either one of 3 mL, 5 mL, and 10 mL syringe in their respective combination with the original needle.

It is well accepted that the difference between PBF and DGF in each syringe and needle combination is dependent upon the needle’s inner diameter, length, and cross-section area of the syringe plunger (8, 9). As the smaller needle diameter would require bigger forces in the use of bigger syringe volume, the clinical application of syringe and needle selection is determined by the least PBF and DGF values. In spite of the fact that the 1 mL syringe poses the least forces needed to create the least pain in the clinical setting, it bears the smallest volume of injection. While maintaining the needle in the site of a puncture in a stable position, injection using 1 mL syringe would unquestionably need multiple reloads to reach the volume of injection when more than the 1 mL solution is needed. Assistance would undeniably be needed to help with the syringe reloads.

This experimental study could not be just simply inclusive in the clinical practice. Similar to our study, previous in-vitro and in-vivo study, which was quite complex and focused on the scoring system to rationalise and support the selection of optimal needle’s diameter and length, does not provide clinically practicable findings (13). A well designed RCT study would be following this research to tackle the influencing factors for pain in the clinical setting.

## Conclusion

Based on the lowest plunger-stopper break loose force and dynamic gliding force, the best-recommended combination to perform local injection to reduce pain is a 1 mL syringe and the original 26G needle. It could be also modified with the use of a 27G spinal or 27G needles. A video about a clinical example on how the local anesthesia was performed for FAHS full awake hand surgery using the 1 mL syringe paired with a 26G needle is supplemented (Video 1).

## Supplementary Information

**Table 1 Suppl:** Needle and syringe characteristics

Variables	Diameter (mm)	Length (mm)
Needle characteristics		
26G needle (original from 1 mL syringe)	0.45	13
23G needle (original from 3 mL syringe)	0.65	32
22G needle (original from 5 mL syringe)	0.7	38
21G needle (original from 10 mL syringe)	0.8	38
27G needle	0.4	13
27G spinal needle	0.42	88
30G needle	0.3	13
Syringe characteristics		
1 mL syringe	OD 6.75; ID 4.75	72.1
3 mL syringe	OD 10; ID 9.15	60
5 mL syringe	OD 14.05; ID 13.3	58.5
10 mL syringe	OD 17.2; ID 16.15	75
20 mL syringe	OD 21.7; ID 20.3	93.45

OD (outer diameter), ID (inner diameter)

**Table 2 Suppl:** PBF and DGF of syringe and needle combinations

Syringe	Needle		PBF (N)		DGF (N)	
		
	No	Length (mm)	Mean (SD)	95%CI	Mean (SD)	95%CI
1 mL	ORI	13	1.15 (0.22)	[0.61–1.68]	0.13 (0.05)	[0.00–0.25]
	27GN	13	0.87 (0.21)	[0.35–1.38]	0.31 (0.00)	[0.31–0.31]
	27GSN	88	0.75 (0.087)	[0.53–0.97]	0.36 (0.10)	[0.34–0.39]
	30GN	13	0.79 (0.10)	[0.54–1.04]	0.71(0.06)	[0.55–0.87]
3 mL	ORI	32	1.95 (0.13)	[1.62–2.28]	1.33 (0.153)	[0.75–1.51]
	27GN	13	2.60 (0.54)	[1.27–3.93]	3.04 (0.43)	[2.94–3.15]
	27GSN	88	4.37 (0.058)	[4.22–4.5101]	4.56 (0.05)	[4.44–4.67]
	30GN	13	4.83 (0.59)	[3.37–6.30]	11.48 (0.45)	[10.36–12.60]
5 mL	ORI	38	2.33 (0.29)	[1.60–3.05]	1.83 (0.056)	[1.70–1.97]
	27GN	13	10.42 (0.52)	[9.12–11.71]	13.50 (0.00)	[13.50–13.50]
	27GSN	88	10.56 (0.96)	[8.17–12.95]	19.50 (0.00)	[19.50–19.50]
	30GN	13	12.47 (0.99)	[10.02–14.92]	58.53 (0.46)	[57.39–59.68]
10 mL	ORI	38	3.27 (0.32)	[2.47–4.07]	1.88 (0.48)	[0.70–3.06]
	27GN	13	4.73 (0.46)	[3.59–5.88]	36.24 (0.58)	[34.80–37.67]
	27GSN	88	4.33 (0.58)	[2.90–5.77]	42.89 (0.77)	[40.98–44.79]
	30GN	13	46.07 (20.15)	[−4.00–96.13]	177.83 (3.26)	[169.73–185.94]
20 mL	27GN	13	28.33 (1.44)	[24.75–31.92]	134.53 (0.61)	[133.02–136.05]
	27GSN	88	25.33 (0.58)	[23.90–26.77]	113.27 (1.55)	[109.41–117.13]
	30GN	13	N/A	–	N/A	–

DGF (dynamic gliding force), N (Newtons), ORI (original needle from packaging of the syringe), PBF (plunger-stopper break-loose force), 27GN (27 Gauge needle), 27GSN (27 Gauge spinal needle), 30GN (30 Gauge needle)

## Figures and Tables

**Video 1:** FAHS to support injection kit 1. This video can be watched by clicking on to the figure or on YouTube at https://youtu.be/6X0NManP1wc

**Table 1:** Statistical analysis for PBF and DGF of various syringe and needle combinations

			PBF (N)			DGF (N)				
	
			Mean difference	95%CI	*P*-value^b^	*F*-statistic (df1,df2)^a^;*P*-value^a^	Mean difference	95%CI	*P*-value^b^	*F*-statistic (df1,df2)^a^;*P*-value^a^
1 mL syringe										
ORI	versus	27GN	0.28	−0.19, 0.75	0.420	*F*(3, 8) = 3.545; *P* = 0.068	−0.19	−0.30, −0.07*	0.003	*F*(3, 8) = 103.881; *P* = 0.000
ORI	versus	27GSN	0.40	−0.07, 0.86	0.109		−0.23	−0.35, −0.12*	0.001	
ORI	versus	30GN	0.35	−0.11, 0.82	0.179		−0.58	−0.70, −0.46*	0.000	
27GN	versus	27GSN	0.12	−0.35, 0.58	1.000		−0.05	−0.16, 0.07	1.000	
27GN	versus	30GN	0.07	−0.39, 0.54	1.000		−0.39	−0.51, −0.28*	0.000	
27GSN	versus	30GN	−0.04	−0.51, 0.42	1.000		−0.35	−0.46, −0.23*	0.000	
3 mL syringe										
ORI	versus	27GN	−0.65	−1.80, 0.50	0.510	*F*(3, 8) = 34.845; *P* < 0.001	−1.91	−2.59, −1.23*	0.000	*F*(3, 8) = 1051.939; *P* < 0.001
ORI	versus	27GSN	−2.42	−3.57, −1.27*	0.001		−3.42	−4.11, −2.74*	0.000	
ORI	versus	30GN	−2.88	−4.04, −1.73*	0.000		−10.35	−11.03, −9.66*	0.000	
27GN	versus	27GSN	−1.77	−2.92, −0.62*	0.004		−1.51	−2.20, −0.83*	0.000	
27GN	versus	30GN	−2.23	−3.38, −1.08*	0.001		−8.44	−9.12, −7.75*	0.000	
27GSN	versus	30GN	−0.47	−1.62, 0.69	1.000		−6.93	−7.61, −6.24*	0.000	
5 mL syringe										
ORI	versus	27GN	−8.09	−10.22, −5.96*	0.000	*F*(3, 8) = 108.173; *P* < 0.001	−11.67	−12.33, −11.01*	0.000	*F*(3, 8)= 33503.958; *P* < 0.001
ORI	versus	27GSN	−8.23	−10.36, −6.10*	0.000		−17.67	−18.33, −17.00*	0.000	
ORI	versus	30GN	−10.14	−12.27, −8.01*	0.000		−56.70	−57.36, −56.04*	0.000	
27GN	versus	27GSN	−0.14	−2.27, 1.99	1.000		−6.00	−6.66, −5.34*	0.000	
27GN	versus	30GN	−2.05	−4.18, 0.08	0.061		−45.03	−45.69, −44.37*	0.000	
27GSN	versus	30GN	−1.91	−4.04, 0.22	0.086		−39.03	−39.69, −38.37*	0.000	
10 mL syringe										
ORI	versus	27GN	−1.47	−30.11, 27.18	1.000	*F*(3, 8)= 12.992; *P* = 0.002	−34.36	−39.24, −29.48*	0.000	*F*(3, 8)= 6114.772; *P* < 0.001
ORI	versus	27GSN	−1.07	−29.71, 27.58	1.000		−41.01	−45.88, −36.13*	0.000	
ORI	versus	30GN	−42.80	−71.45, −14.15*	0.005		−175.95	−180,83, −171.08*	0.000	
27GN	versus	27GSN	0.40	−28.25, 29.15	1.000		−6.65	−11,53, −1.77*	0.009	
27GN	versus	30GN	−41.33	−70.00, −12.69*	0.006		−141.60	−146.47, −136.72*	0.000	
27GSN	versus	30GN	−41.73	−70.38, −13.09*	0.006		−134.95	−139.82, −130.07*	0.000	
27GN	versus	27GSN	3.00	0.58, 5.42*	0.020	*F*(2, 6) = 766.569; *P* < 0.001	21.27	18.68, 23.86*	0.000	*F*(2, 6) = 16666.452; *P* < 0.001

* *P* < 0.05,

a One-way ANOVA,

b Post-hoc analysis with Bonferroni corrections

DGF (dynamic gliding force), N (Newtons), ORI (original needle from packaging of the syringe), PBF (plunger-stopper break-loose force), df1 (the numerator degree of freedom), df2 (the denominator degree of freedom), 27GN (27 Gauge needle), 27GSN (27 Gauge spinal needle), 30GN (30 Gauge needle)

**Table 2:** Statistical analysis for PBF and DGF of original pairs of needle and syringe combinations

			PBF(N)				DGF(N)			
	
			Mean difference	95%CI	*P*-value^b^	*F*-statistic (df1,df2)^a^; *P*-value^a^	Mean difference	95%CI	*P*-value^b^	*F*-statistic (df1,df2)^a^; *P*-value^a^
Original needle										
1 mL	versus	3 mL	−0.80	−1.48, −0.12*	0.018	(4, 10) = 32.403; *P* < 0.001	−1.01	−1.67, −0.35*	0.003	(4, 10) = 32.752; *P* < 0.001
1 mL	versus	5 mL	−1.18	−1.86, −0.50*	0.001		−1.71	−2.37, −1.05*	0.000	
1 mL	versus	10 mL	−2.12	2.81, −1.44*	0.000		−1.75	−2.42, −1.10*	0.000	
3 mL	versus	5 mL	−0.38	−1.06, 0.31	0.765		−0.70	−1.36, −0.04*	0.036	
3 mL	versus	10 mL	−1.32	−2.00, −0.64*	0.000		−0.75	−1.41, −0.09*	0.023	
5 mL	versus	10 mL	−0.94	−1.62, −0.26*	0.006		−0.05	−0.71, 0.61	1.000	

* *P* < 0.05,

a One-way ANOVA,

b Post-hoc analysis with Bonferroni corrections

DGF (dynamic gliding force), N (Newtons), PBF (plunger-stopper break-loose force), df1 (the numerator degree of freedom), df2 (the denominator degree of freedom)
